# ID 177 - Análise *Ex Ante* da Rede Brasileira de Pesquisa Clínica

**DOI:** 10.5327/2237-9622.2025.v34s1.177

**Published:** 2025-11-25

**Authors:** Andrea Leite Ribeiro, Giovanny Vinícius Araújo de França, Raísa Breda Tôso Sfalsini, Ana Carolina de Castro Nobre, Michelle Zanon Pereira, Evandro de Oliveira Lupatini, Monica Felts de La Roca Soares

**Keywords:** rede, pesquisa clínica, análise *ex ante*

## Abstract

**Introdução:**

As políticas públicas compreendem um conjunto de ações e diretrizes estatais voltadas à solução de problemas e à necessidades da sociedade. A avaliação, por meio da análise, é um componente que deve estar presente desde o nascedouro das políticas públicas, para otimizar os recursos públicos e o bem-estar da população. A análise constitui uma avaliação destinada a identificar inconsistências e incoerências em aspectos para a criação de uma política pública. Nesse contexto, objetiva-se apresentar os resultados da análise da instituição da Rede Brasileira de Pesquisa Clínica (RBPClin), no âmbito do governo federal.

**Método:**

No período de outubro/2022 a novembro/2023, foi realizada a análise da RBPClin, a partir da metodologia definida pelo, da Casa Civil (Brasil, 2018), com vistas a desenvolver ferramentas e análises para estruturar a rede. O trabalho foi conduzido pela Coordenação-Geral de Evidências em Saúde, em parceria com a Coordenação-Geral de Ações Estratégicas em Pesquisa Clínica, ambas do Departamento de Ciência e Tecnologia da Sectics/MS. Foram realizadas três oficinas com a equipe técnica responsável pela implementação da RBPClin e estudo de documentos sobre o tema. A análise seguiu as etapas de: diagnóstico do problema; caracterização da política: objetivos, ações, público-alvo e resultados esperados; desenho da política; estratégia de construção de confiabilidade e de credibilidade; estratégia de implementação; e estratégia de monitoramento, de avaliação e de controle.

**Resultados:**

A RBPClin surgiu como resposta ao Plano de Ação de Pesquisa Clínica no Brasil, institucionalizado por meio da Portaria GM/MS n.º 559/2018. Como ações estratégicas, o Plano propôs a reestruturação do modelo de gestão da Rede Nacional de Pesquisa Clínica (RNPC) e o fortalecimento do trabalho colaborativo em rede. À época, compreendia-se que o modelo da RNPC limitou o alcance do seu pleno potencial, culminando com a concepção de uma nova rede. A análise foi iniciada ainda na fase de concepção da RBPClin. Os principais achados referem-se à elaboração do diagnóstico do problema, à árvore do problema e aos objetivos da Rede. Já em relação ao desenho e à implementação da intervenção, destacam-se a elaboração do modelo lógico, da teoria da mudança e da matriz de indicadores; a análise das políticas relacionadas e atores sociais; e o mapeamento dos riscos utilizando a Matriz Swot, identificando possíveis meios para superá-los a partir da Matriz Swot Cruzada. Por fim, realizou-se a análise do grau de confiança e suporte da intervenção.

**Conclusão:**

A análise foi essencial para subsidiar a implementação da RBPClin de maneira eficaz. A capacidade limitada de execução de pesquisa clínica no âmbito nacional e a alta concentração regional da capacidade instalada constituem os principais problemas a serem enfrentados pela Rede. Para solucionar esses problemas, a RBPClin deve atuar para promover a articulação entre pesquisadores, especialistas, organizações e instituições no âmbito da pesquisa clínica, apoiar a formação e a capacitação em pesquisa clínica, e fomentar a qualificação técnica e a infraestrutura de centros de pesquisa clínica. Conclui-se, destacando-se a relevância da análise da RBPClin, por prover subsídios fundamentais para a avaliação contínua de sua atuação frente aos desafios enfrentados pelo ecossistema da pesquisa clínica no País.

